# Effect of microfluidic processing on the viability of boar and bull spermatozoa

**DOI:** 10.1063/5.0013919

**Published:** 2020-08-03

**Authors:** Tanja Hamacher, Johanna T. W. Berendsen, Stella A. Kruit, Marleen L. W. J. Broekhuijse, Loes I. Segerink

**Affiliations:** 1 BIOS Lab on a Chip Group, MESA+ Institute for Nanotechnology, Technical Medical Centre, Faculty of Electrical Engineering, Mathematics and Computer Science, University of Twente, P.O. Box 217, 7500 AE Enschede, The Netherlands; 2 CRV BV, Wassenaarweg 20, 6843 NW Arnhem, The Netherlands; 3 Topigs Norsvin Research Center B.V., P.O. Box 43, 6640 AA, Beuningen, The Netherlands

## Abstract

The use of microfluidics in artificial reproductive technologies for manipulation or assessment of spermatozoa is unique in the sense that it is not always an end point measurement and the sample may be used afterward. During microfluidic processing, spermatozoa are exposed to shear stress, which may harm viability and functioning of spermatozoa. The shear stresses during general microfluidic processing steps were calculated and compared to estimated shear stresses during ejaculation. The viability of boar and bull spermatozoa after microfluidic processing was studied and compared to the typical handling method (centrifugation) and to a control (the sample in a tube at the same temperature). The boar spermatozoa showed a small but significant decrease in viability of 6% after microfluidic handling. Bull spermatozoa proved to be less susceptible to shear stress and were not significantly affected by microfluidic processing. These data indicate that the impact of microfluidic processing on the viability of boar and bull spermatozoa is less than the literature values reported for flow cytometry and comparable to the impact of centrifugation.

## I. INTRODUCTION

Artificial reproductive technologies (ART), such as artificial insemination (AI), are commonly used to support the mechanism of fertilization, both for couples with fertility problems and in the veterinary industry.^1^ Approximately 9% of all couples in developed countries have infertility problems, of which 56% are looking for medical care.^2^ In the veterinary industry, professional farms worldwide breed approximately 90% of pigs and 80% of dairy cattle using AI.^1^ For example, for pork production, the European Union and the USA use 95% and 90% AI, respectively.^3^ Part of the success of ART is determined by semen quality. Poor quality spermatozoa, such as morphologically abnormal or immotile spermatozoa, and the presence of external substances, for example, other cells, debris, and micro-organisms, reduce the success rate of ART.^4^

To control the success rate of ART in the veterinary industry, semen quality assessment is performed by determining semen characteristics such as sperm count, morphology, and motility. The success rate of ART in clinics and veterinary industry can be increased by improving the quality of the sample by selecting only “good” spermatozoa. Established techniques in clinics and/or veterinary industry include among others computer-assisted sperm analysis (CASA),^5,6^ flow cytometry,^7,8^ density gradient centrifugation,^9,10^ and swim-up.^10,11^ However, these techniques are time consuming, expensive, and require trained personnel.

Microfluidics is a fast-emerging field, dealing with the flow of liquids inside micrometer-sized channels, which match the size range of cells. Microfluidics can provide advantages over conventional semen processing techniques such as standardization, low costs, and ease of visualization. In the field of AI, microfluidic devices have been applied to study, analyze, select, and sort spermatozoa.^12–14^ A drawback of microfluidics is the presence of shear stress that is known to reduce mammalian cell viability and affects cell physical and biological properties.^15–18^ Shear stress is defined as the force exerted per unit area by the flowing fluid in a non-uniform velocity field.

In many biomedical applications of microfluidics, such as in blood diagnostics, end point measurements are used. After microfluidic processing, the sample is no longer useful and, therefore, discarded.^19,20^ Hence, the negative effects of microfluidic processing on the cells used for these measurements are not important. However, in the context of ART, viable spermatozoa are needed for successful fertilization. It is essential that the effect on semen quality is minimal while processing, since there is no end point measurement. Only when semen viability and motility after microfluidic processing are preserved, the success rate of ART is retained. Although many microfluidic chips have been proposed to improve semen quality,^12,13,21^ so far no systematic study on the viability of spermatozoa after microfluidic processing has been performed.

Recently, high throughput microfluidic processing of spermatozoa has gained increasing interest. An example is the separation of spermatozoa from erythrocytes using a spiral channel by Son *et al.*^22^ In the spiral channel (150 *μ*m channel width and 50 *μ*m channel height), the motile spermatozoa were forced to the outer channel wall with a flow rate ranging from 0.10 to 0.52 ml/min, where they were exposed to shear stress (estimated to be 27–139 N/m^2^). The separated spermatozoa were, however, not tested for viability or motility. Wu *et al.*^23^ separated spermatozoa of different motilities based on the cell's swimming abilities in a retarding flow field. A flow field (0.3 × 10^−3^ ml/min, estimated shear stress of 0.033 N/m^2^) was needed to carry the spermatozoa to the separation zone. Also, here it is not known whether the spermatozoa processing harmed sperm viability and motility. De Wagenaar *et al.* have developed a chip that focuses spermatozoa using dielectrophoresis (DEP) and sorts morphologically abnormal spermatozoa from normal ones based on the difference in the cells impedance curve.^24^ The preliminary data suggest a minimal effect of DEP on the integrity of the plasma membrane at frequencies above 10 MHz at 3 or 6 V potential. Pinched flow fractionation (PFF) for the separation of spermatozoa from epithelial cells and erythrocytes has been presented by Liu *et al.* and Berendsen *et al.*, respectively.^25,26^ The separation mechanism in PFF is based on the sudden broadening of the channel after the pinched segment.^27^ To achieve separation, it is necessary to align the cells to the sidewall of the pinched segment. Due to the shape, alignment, and flow in the pinched segment, the cells encounter relatively high shear stress compared to other areas of the chip. Berendsen *et al.*^26^ studied the viability after separation with PFF and reported a viability of 88 ± 6% (*n* = 3).

For proof-of-principles, boar spermatozoa are often used as a model for human spermatozoa^28,29^ because human spermatozoa are not widely accessible due to variations in legislation. Moreover, microfluidic processing of spermatozoa has gained attention in the veterinary industry. Li *et al.* and Sano *et al.* have applied a microfluidic sperm sorter based on spermatozoa motility for the selection and production of dairy cattle and porcine embryos, respectively.^30,31^ Their results have shown that *in vitro* fertilization after spermatozoa selection is more successful compared to their control groups. However, for *in vivo* fertilization, separation via the self-motion of spermatozoa is not desired, because the spermatozoa may be exhausted after being separated. Also, the throughput is very limited. In non-motility based separations, a high flow rate is desirable to obtain an acceptable throughput, which exposes the cells to a higher shear stress.

In this study, we test the impact of microfluidic processing on the viability of spermatozoa. Various parts of a microfluidic setup can impose shear stress on spermatozoa. The sample is introduced into the microfluidic chip and collected after microfluidic processing with connection tubing. The amount of shear stress in a microfluidic chip is determined by the dimensions of the channel (cross section) and the flow rate in the chip. In general, the tubing is longer compared to the length of the microfluidic chip, and therefore, the cells are for a longer time exposed to shear stress in the tubing, even though the shear stress in the tubing is commonly lower than in the microfluidic chip due to the larger diameter. Therefore, we have systematically studied the viability of boar and bull spermatozoa after being processed with microfluidic chips and connection tubing. For general purposes, a microfluidic chip with a straight channel was used and as a special case a chip with PFF. Constrictions similar to the pinched segment of a PFF device are used in other microfluidic devices such as flow cytometers and Coulter counters.^29,32,33^ Furthermore, in our investigation, the shear stresses in the tubing and chips used were calculated and compared to shear stress during ejaculation.

## II. MATERIALS AND METHODS

### A. Chip design and fabrication

**FIG. 1.**
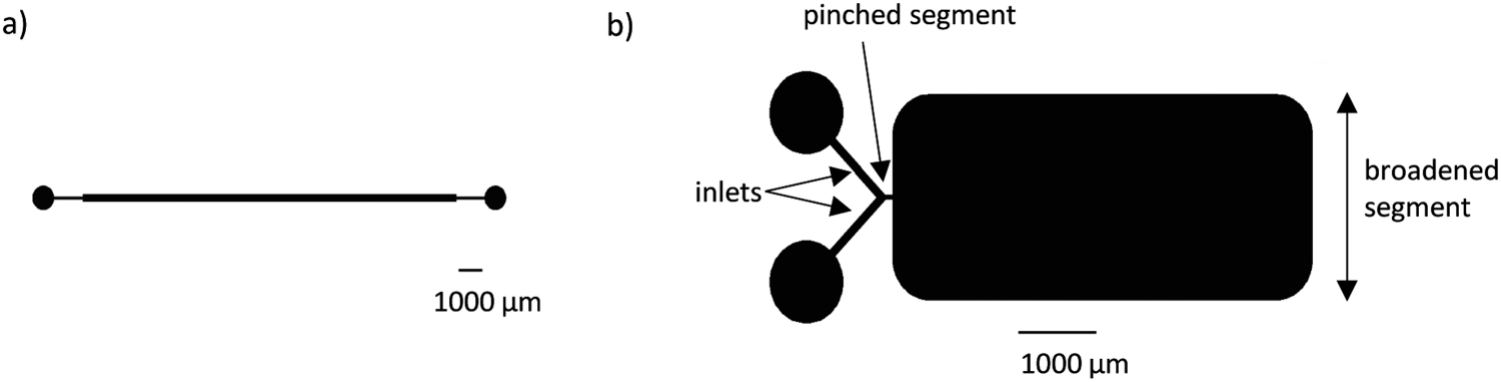
Schematic representation of the microfluidic chips used to process spermatozoa. (a) Straight channel with 300 *μ*m width and 50 *μ*m height (length 2 cm) and (b) PFF chip, with 100 *μ*m wide inlets, 50 *μ*m wide pinched section, and 2500 *μ*m wide broadened section (total length of 8 mm).

Two microfluidic chips were used for the experiments. One chip had a straight channel with 300 *μ*m width and 50 *μ*m height [length (L) 2 cm], and another was a 50 *μ*m high PFF chip, with 100 *μ*m wide inlets, 50 *μ*m wide pinched section, and 2500 *μ*m wide broadened section (total length of 8 mm) (Fig. 1). The chips were designed using CleWin software (version 5.0.12.0). Master molds for polydimethylsiloxane (PDMS) fabrication were produced by standard photolithography. A 50 *μ*m layer of SU-8 (Microchem, Berlin, Germany) was spun and developed on a 4″ silicon wafer.

Chips were fabricated using PDMS (Sylgard 184, Dow Corning, Midland, MI, USA) in a 1:10 v/v ratio of base vs curing agent. PDMS was poured onto a silicon wafer, degassed, and cured at 60 °C overnight. After curing, microfluidic inlets and outlets were punched using a Harris Uni-Core puncher [tip inner diameter (ID) 1.0 mm, Ted Pella Inc., Redding, CA, USA]. The chips were bonded to the glass microscope slides after activation by oxygen plasma using a plasma cleaner (model CUTE, Femto Science, Hwaseong-Si, South Korea).

### B. Sample preparation

Fresh boar semen [breed: Tempo (Topigs Norsvin breeding line), AIM the Netherlands, Vught, the Netherlands] and fresh bull semen (breed: Holstein, CRV, Arnhem, the Netherlands) were obtained at a concentration of 20 × 10^6^ cells/ml and 89 × 10^6^ cells/ml, respectively. Fresh boar semen was stored at 17 °C, and fresh bull semen was stored on ice before use. Before the semen was processed, boar semen was diluted with the Solusem extender (AIM Worldwide, Vught, the Netherlands) to concentrations of 10 × 10^6^ cells/ml (for Group 3) and 4 × 10^6^ cells/ml (for Groups 0–2 and 4). The bull semen was diluted with the Optixcell® extender (IMV technologies, L’Aigle, France) to concentrations of 44 × 10^6^ cells/ml (for Group 3) and 18 × 10^6^ cells/ml (for Groups 0–2 and 4).

### C. Microfluidic/Chip processing

The in- and outlets of the chip were connected to containers using fused silica capillaries [Polymicro Technologies, ID 100 *μ*m, outer diameter (OD) 360 *μ*m, L 10 cm, Molex, Surrey, UK] and Tygon tubing (ND 100-80, ID 250 *μ*m, OD 760 *μ*m, L 20 cm, Saint-Gobain Performance Plastics, Akron, OH, USA). A pressure pump (MZ flows, Fluigent, Le Kremlin-Bicêtre, France) was connected to the sample and buffer containers. The pressure pump was used to apply the flow through the chip.

Shortly before use, the chips were oxygen plasma treated using a plasma cleaner (model CUTE, Femto Science, Hwaseong-Si, South Korea) and became hydrophilic. The chips were rinsed and incubated with poly(l-lysine)-grafted-poly(ethylene glycol) (PLL-g-PEG, SuSoS, Dübendorf, Switzerland) at a concentration of 100 *μ*g/ml in de-ionized (DI) water for at least 15 min. Subsequently, the sample and the buffer solution were introduced. Flow was induced by applying the desired pressures to the sample and buffer solution. At the outlet, the processed sample was collected.

**FIG. 2.**
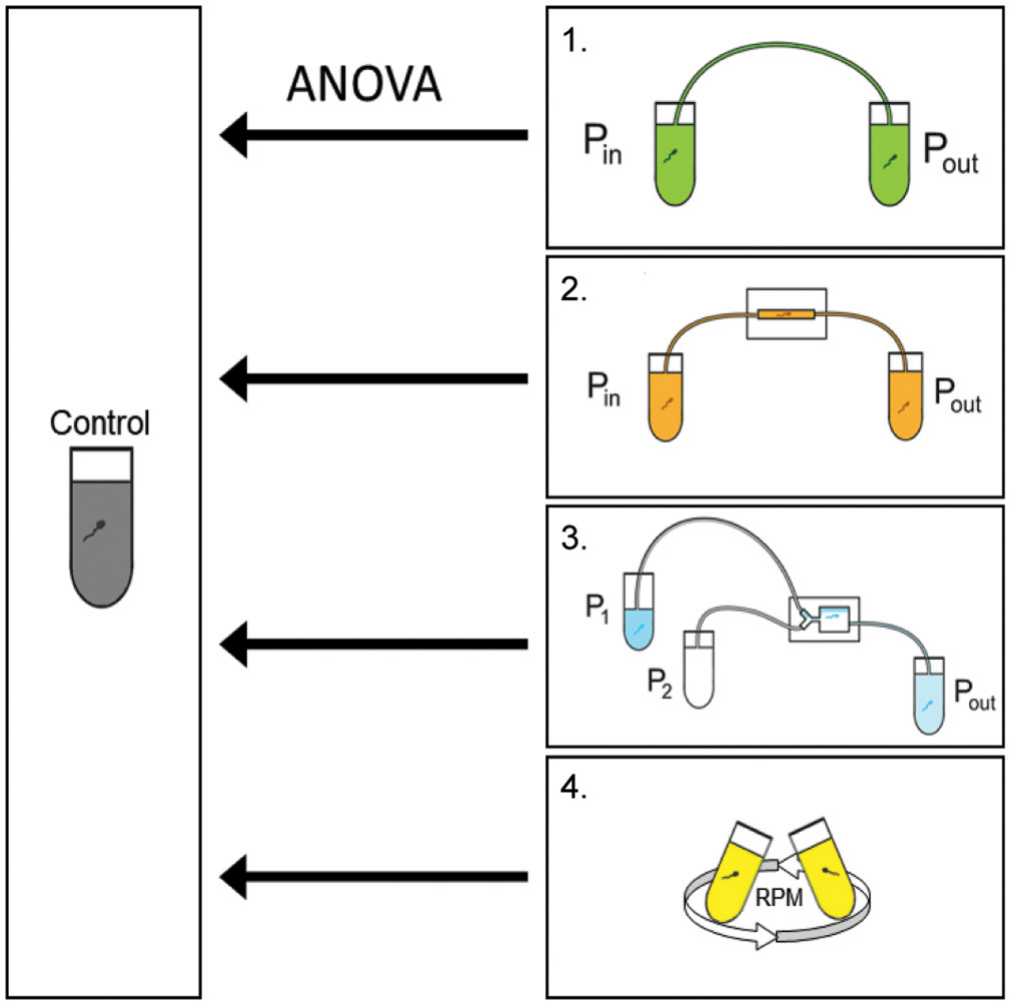
Schematic overview of the three different tested conditions for microfluidic processing of (1) tubing, (2) straight channel, (3) PFF channel, and (4) centrifuge for both bull and boar spermatozoa. P_in_ [or P_1_ (sample) and P_2_ (buffer)] was varied, while P_out_ was kept at atmospheric pressure. Each sample group was compared to the control with ANOVA to determine the significance of the change in viability. Features are not to scale. Pressure (P).

Four experimental setups were tested (see Fig. 2 and Table I): (1) a set with only the tubing connected (applied pressures: 200, 400, 600, 800, and 1000 mbar; boar semen sample concentration: 4 × 10^6^ cells/ml; bull semen sample concentration: 18 × 10^6^ cells/ml), (2) a straight microfluidic channel with in- and outlet tubing (applied pressures: 200, 400, 600, 800, and 1000 mbar; boar semen sample concentration: 4 × 10^6^ cells/ml; bull semen sample concentration: 18 × 10^6^ cells/ml), (3) a pinched flow channel with in- and outlet tubing (sample/sheath pressures in mbar: 200/200, 200/300, 400/400, 400/600, 600/600, and 600/800; boar semen sample concentration: 10 × 10^6^ cells/ml; bull semen sample concentration: 44 × 10^6^ cells/ml), and (4) centrifugal forces at different speeds for 15 min using the Minispin Plus (Eppendorf, Hamburg, Germany) {relative centrifugal forces (RCF): 700, 1500, and 3000 × g [3230, 4729 and 6688 rounds per minute (rpm)]; boar semen sample concentration: 4 × 10^6^ cells/ml; bull semen sample concentration: 18 × 10^6^ cells/ml}. All experiments were performed at room temperature to minimize the swimming behavior of the spermatozoa. Each setup was assessed three times with the same conditions (N = 3). Exceptions (N = 2) are the experiments performed with boar semen and a straight channel (pressure: 600 mbar) as well as boar semen and the pinched flow channel (sample/sheath pressures in mbar: 200:300 and 400:600), because the third measurements counted less than 200 spermatozoa and was, therefore, excluded. For the centrifugation of boar spermatozoa, a different control sample was used than for the other experimental setups.

**TABLE I.** Calculated shear stress and its duration during boar and bull ejaculation as well as during microfluidic processing with tubing and a chip with a straight channel. For both ejaculation and microfluidic processing, the shear stresses were in the same order of magnitude. The highest shear stress occurred in the capillary part of the tubing or in the pinched segment of the PFF chip. The longest duration was in the Tygon tubing, where the shear stress was low.

		Flow rate (ml/min)	Shear stress (N/m^2^)	Duration time (s)
Boar ejaculation		40	3.4	3.6
Bull ejaculation		1.2 × 10^2^	0.65	9.8
Flow cytometer			2.2–79	≈few seconds
Microfluidics	Pressure (mbar)	Total flow rate (ml/min)	Peak shear stress (N/m^2^) [duration time (s)]	Longest exposure shear stress (N/m^2^) [duration time (s)]
Tubing	200	2.8 × 10^−2^	4.8 (1.7)	0.30 (21)
	400	5.6 × 10^−2^	9.5 (0.84)	0.61 (11)
	600	8.4 × 10^−2^	14 (0.56)	0.91 (7.0)
	800	0.11	19 (0.42)	1.2 (5.3)
	1000	0.14	24 (0.34)	1.5 (4.2)
Chip with straight channel	200	4.0 × 10^−3^	2.2 (7.3)	0.17 (9.1)
	400	7.8 × 10^−3^	4.4 (3.6)	0.28 (4.6)
	600	1.2 × 10^−2^	6.6 (2.4)	0.42 (3.0)
	800	1.6 × 10^−2^	8.8 (1.8)	0.56 (2.3)
	1000	2.0 × 10^−2^	11 (1.5)	0.70 (1.8)
PFF chip	200/200	1.8 × 10^−2^	15 (8.3 × 10^−4^)	0.20 (33)
	200/300	2.3 × 10^−2^	18 (6.6 × 10^−4^)	0.25 (26)
	400/400	3.7 × 10^−2^	29 (4.1 × 10^−4^)	0.39 (16)
	400/600	4.6 × 10^−2^	36 (3.3 × 10^−4^)	0.49 (13)
	600/600	5.5 × 10^−2^	44 (2.8 × 10^−4^)	0.59 (11)
	600/800	6.7 × 10^−2^	51 (2.4 × 10^−4^)	0.69 (9.3)

### D. Viability staining

The influence of shear stress from the tubing and the chips on the viability of the spermatozoa was assessed with a SYBR 14/Propidium Iodide (PI) live/dead staining. The spermatozoa were incubated in a 1000× dilution of SYBR 14 (stock 1 mM, ex/em 488/518 nm, Life Technologies, Eugene, OR, USA) for 20 min and in a 100× dilution of PI (stock 2.4 mM, ex/em 535/617 nm, Life Technologies, Eugene, OR, USA) for 5 min at room temperature. For each sample, 20 *μ*l was deposited onto a glass slide. Images were taken with an EVOS M5000 (ThermoFisher Scientific, Waltham, MA, USA). The number of live and dead cells was manually counted, and the percentage of live cells was determined by dividing the number of live cells by the total number of cells. Per sample, at least 200 spermatozoa were counted to have statistically relevant data.^21^ To obtain the effect of the chip on the viability of the cells, the percentage of viable spermatozoa in the processed samples was normalized by dividing it by the percentage of viable spermatozoa of the control sample (the diluted spermatozoa sample was kept in the container and was not processed).

### E. Statistical analysis

The normal distribution was tested using the Shapiro–Wilk test, which is an appropriate test for small sample sizes. A one-way, between-groups analysis of variance (ANOVA) with planned comparisons was conducted to explore the effect of microfluidic processing on the viability of spermatozoa. An ANOVA analysis compares the variances between the different groups. A between-groups ANOVA is applied when different participants are present. In this study, the “participants” are the individual experimental conditions (*n* = 2–6). The effects of various microfluidic processing procedures are compared to the control group. Therefore, a planned comparison was used to overcome “power” issues.^34^ A positive one-tail test is more powerful in this context, as it is impossible to achieve higher viability after microfluidic processing.

The samples were divided into five groups according to the experiment (Group 0: control; Group 1: tubing; Group 2: chip with straight channel; Group 3: PFF chip; and Group 4: centrifugal forces). The groups obtained after microfluidic processing (Groups 1–3) and after exposure to centrifugal forces (Group 4) were compared with the control group (Group 0) (Fig. 2). The significance level was chosen to be 0.05.

## III. RESULTS AND DISCUSSION

### A. Flow and shear stress calculations

The wall shear stresses during ejaculation and the microfluidic processing as performed in our study were calculated. Detailed information about the calculations can be found in the supplementary material (see S.1). Table I shows the calculated flow rate and wall shear stresses during ejaculation, flow cytometry, and microfluidic processing. The peak shear stress represents the highest shear stress of each experiment. The duration is the time the cells were exposed to the peak shear stress.

During ejaculations, the Reynolds numbers do not exceed 2000, so the flow is laminar. The spermatozoa were exposed to a wall shear stress of 3.4 N/m^2^ and 0.65 N/m^2^ during boar and bull ejaculation, respectively. The highest shear stress in the tubing experiments (up to 24 N/m^2^) was induced by the capillary and its small surface area. The shear stress induced by the Tygon tubing was small (up to 1.5 N/m^2^) compared to the highest shear stress, but the spermatozoa were exposed to this shear stress for a longer time period. Depending on the applied pressure and the resulting flow rate, the wall shear stress during microfluidic processing with a straight channel varied from 0.17 to 11 N/m^2^. These shear stresses occurred in the connection tubing rather than in the chip itself. The shear stress in the chip varied between 1.7 and 8.6 N/m^2^, which was lower than the shear stress in the capillary. Both wall shear stresses during ejaculation and microfluidic processing were in the same order of magnitude. The wall shear stress in the pinched section of the PFF device (15–51 N/m^2^) was a magnitude higher than the wall shear stress of the tubing and the straight channel. This resulted from the smaller channel width of the pinched section compared to the tubing diameter and width of the straight channel. Although the wall shear stress in the PFF device was high, the duration of this wall shear stress was short (less than a millisecond) compared to the other durations of a few seconds.

In another comparison, we have calculated the shear stress during flow cytometry. In the veterinary industry, flow cytometry is used to sort spermatozoa to obtain sex selected semen.^35^ Therefore, the cells must be viable after being sorted by flow cytometry. The normalized spermatozoa viability after flow cytometry reported in the literature is 89 ± 3%^36^ and 80 ± 3%^37^ (for boar and bull, respectively). We have estimated the shear stress in flow cytometers to be between 2.2 and 79 N/m^2^ (see supplementary material for estimations on processing velocities and dimensions), which is in the same range as the shear stress of microfluidic processing.

### B. Viability of spermatozoa after microfluidic processing

**FIG. 3.**
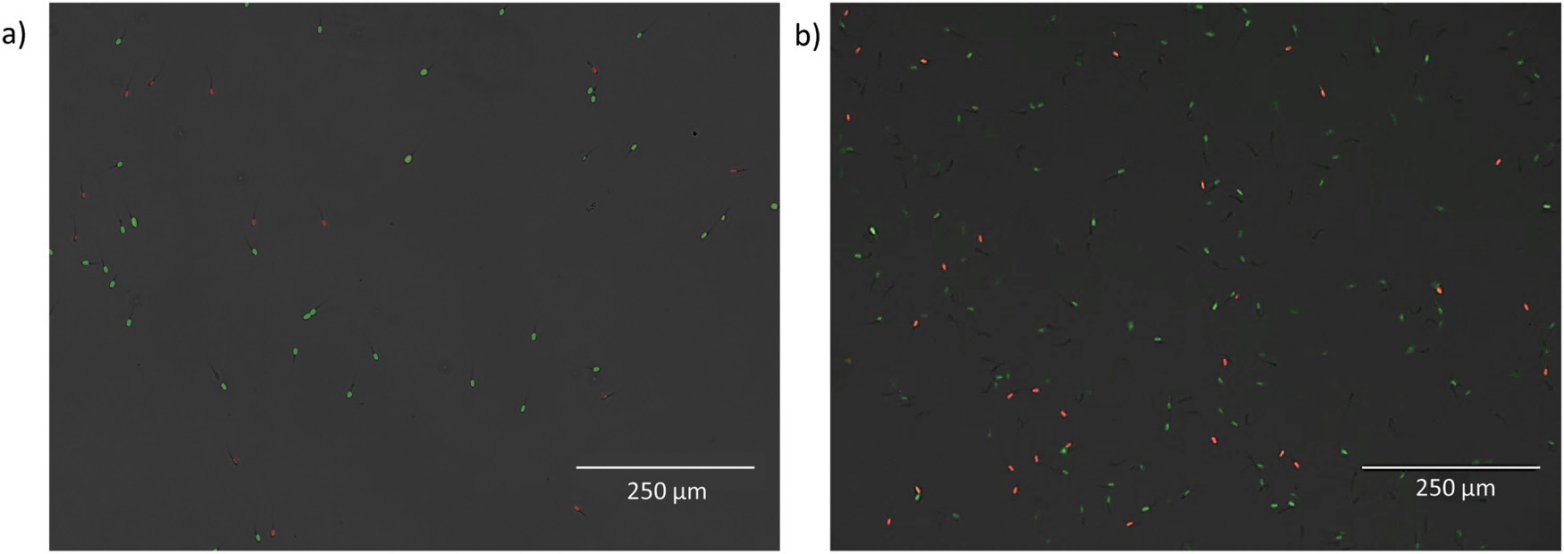
Representative images of boar (a) and bull (b) spermatozoa treated with live/dead staining after being processed with the microfluidic chip (straight channel, 1000 mbar). Live and dead cells are represented in green and red, respectively.

**FIG. 4.**
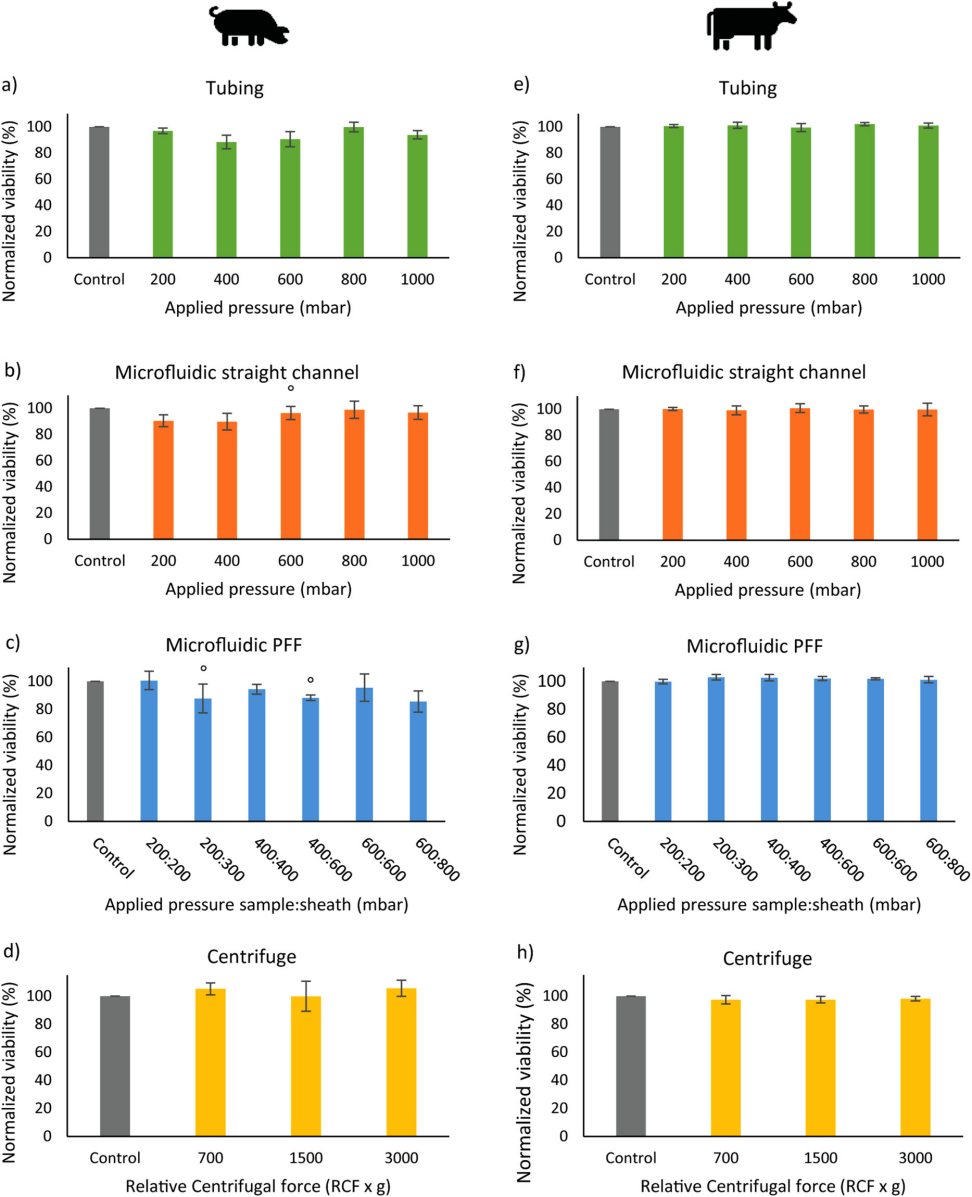
The percentage of normalized viability after microfluidic processing of boar (left) and bull (right) spermatozoa with connection to tubing (a) and (e), the microfluidic chip with a straight channel (b) and (f), a pinch flow fractionation (PFF) chip (c) and (g), and centrifugation (d) and (h). Error bars = 1 SD, N = 3 (° N = 2). In all experimental conditions, no trend in viability decrease with increasing applied pressure/centrifugal force was observed (p > 0.05).

**FIG. 5.**
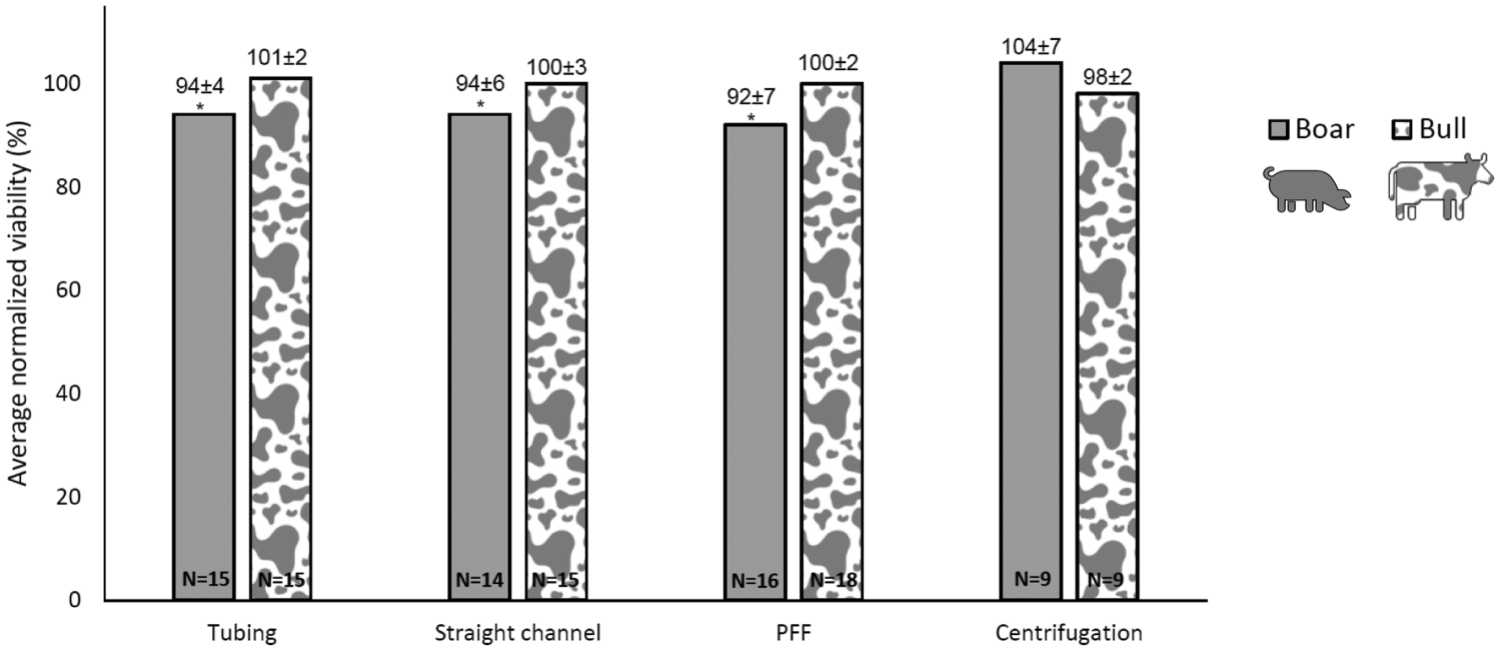
Average normalized viability (±1 SD) of the boar and bull spermatozoa after (microfluidic) processing. The viability of spermatozoa after microfluidic processing was almost 100% when taking the standard deviation into account. Therefore, the effect of microfluidic processing on the viability of spermatozoa is negligible. * indicates p < 0.05.

The effect of microfluidic processing on the viability of spermatozoa was studied by processing spermatozoa with various parts of a microfluidic setup, namely, the connection tubing, a microfluidic chip with a straight channel, and a microfluidic chip with PFF. Various pressures were applied while running the spermatozoa through the systems. Figure 3 shows representative images of live/dead stained spermatozoa after microfluidic processing. The viability of the control group was 83 ± 5.8% for boar spermatozoa and 88 ± 3.1% for bull spermatozoa. In Fig. 4, the normalized viability of bull and boar spermatozoa after microfluidic processing with various applied pressures and centrifugation with various centrifugal forces is shown. It was investigated whether an increase in applied pressure or centrifugal force decreases viability. This possible trend was not observed. Under all four experimental conditions, the viabilities were similar to the other test conditions. For that reason, the normalized average viabilities for each experimental condition were determined (Fig. 5).

The normalized averaged viabilities of the boar and bull spermatozoa were between 88%–98% and 97%–103%, respectively, and were very similar to the control group (100%). This also held for PFF, where the spermatozoa were exposed to the highest shear stress. The lowest viability of 88% viability in boar semen was still high. When taking the standard deviation into account, at first sight it appeared that the effect of the microfluidic chips is negligible. Specifically, the bull semen seemed not to be affected by the microfluidics processing. The results, however, suggest that boar semen is more susceptible to processing than bull semen. Similarly, it has been shown that cryopreservation causes more negative effects on boar than on bull spermatozoa.^38,39^ Differences in physiochemical and biochemical semen characteristics between these animal species may be the reason for this unequal susceptibility.^40^

To find additional evidence to visual observations, the statistical analysis was performed. The Shapiro–Wilk test showed that the normalized viabilities showed a normal distribution (p > 0.05). An ANOVA with planned comparison was applied to test whether there was an impact on viability after microfluidic processing compared to the control group. For boar spermatozoa, viability in the groups with microfluidic processing was significantly (F_1,47_ = 5.12, p = 0.014) lower than the control group. The average decrease in viability of 6% seemed, therefore, to be significant. Only the difference for the control with group 5 (centrifugal forces) was not statistically significant (F_1,13_ = 1.13, p = 0.15). There was no significant difference in viability between processing with tubing, a straight channel, or PFF (p = 0.41). In each of these sets of experiments, the semen was flushed through the tubing to reach the chip. These results could indicate that the spermatozoa are damaged in the tubing before reaching the chip. Therefore, the effect of microfluidic tubing processing has been investigated by varying tubing parameters such as tubing type and length [see supplementary material (see S.2)]. The results show that tubing had no visual nor statistical effect on spermatozoa viability. The average decrease in viability to 94 ± 7% was lower than the decrease in viability when using a flow cytometer as reported in the literature, where normalized viability after sorting was 89 ± 3%.^36^ Note that this decrease in viability could be affected by the biological variation between species and our relatively small sample size.

For bull spermatozoa, the groups with microfluidic processing did not differ significantly (F_1,67 _= 0.09, p = 0.38) from the control group. This indicates that the viability of the bull spermatozoa was not affected by microfluidic processing. In this study, viability was higher than in a study using flow cytometry. The value for the normalized viability (calculated from the absolute viability reported for “bulk sorting,” in which all spermatozoa were counted) was 80 ± 3%.^37^

In contrast to viability measurements, the motility and morphology of spermatozoa provide important information about spermatozoa condition. Observations have shown that after processing, the spermatozoa were intact and showed no morphological difference compared to the control group. To prevent inter-animal differences from occluding the viability results, we have used one semen donor. It is unknown whether these semen donors were representative for the population, but the control semen was collected from a boar and a bull that are used in routine semen processing for AI. Both donors have high fertility recording (Topigs Norsvin, Vught, the Netherlands; CRV, Arnhem, The Netherlands). For further research, it is recommended to test various semen donors and to quantify the motility and morphology of spermatozoa after microfluidic processing.

## IV. CONCLUSIONS

Over the years, microfluidic analysis and processing of spermatozoa have gained more interest. For the intended applications, it is essential that the spermatozoa are not damaged by processing and remain viable for insemination. We estimated the shear stress on bull and boar spermatozoa during ejaculation and compared it to the calculated shear stress during general microfluidic processing steps. The shear stress is comparable to the natural shear stress during ejaculation. We then studied the viability of spermatozoa after microfluidic processing. The boar spermatozoa showed a small but significant decrease in viability of 6%. Bull spermatozoa revealed to be less susceptible; it was concluded that it is not significantly affected by microfluidic processing. These data indicate that microfluidic processing has less influence on the viability of boar and bull spermatozoa than literature has reported for flow cytometry.

## SUPPLEMENTARY MATERIAL

See the supplementary material for the complete description of the flow and shear stress calculations (S.1) and for the results for the effect of microfluidic tubing processing on the spermatozoa viability (S.2).

## AUTHORS’ CONTRIBUTIONS

T.H., J.B., and S.K. equally contributed by designing the study, carrying out the experiments, performing calculations, and writing the manuscript. M.B. organized the semen samples. M.B. and L.S. supervised the project. All authors discussed the results and commented on the manuscript.

## DATA AVAILABILITY

The data that support the findings of this study are available from the corresponding author upon reasonable request.
